# A review of a translational sociology: interdisciplinary perspective on politics and society

**DOI:** 10.3389/fpsyg.2023.1189023

**Published:** 2023-10-31

**Authors:** Jiayan Xiao, Yang Bai, Chili Li

**Affiliations:** ^1^School of Foreign Languages, Hubei University of Technology, Wuhan, China; ^2^Independent Researcher, Wuhan, China

**Keywords:** translation, society, politics, experience, translational sociology

## Abstract

*A Translational Sociology—Interdisciplinary Politics and Society* by Esperança Bielsa in 2023 has aroused our great interest, as the title has explicitly indicated a further fulfillment and development of Translational Sociology from the perspective of interdisciplinary politics and society. The recent decade witnesses how Translational Sociology is well-received in China, the further exploration of translation and society, translation and politics, translation and experience in this book enables more teachers, researchers, and postgraduates in translation study to be fully aware of the diversity, complexity, and modernity of translation as a social practice. Moreover, by reinforcing the core role of translation in mediating language differences in all aspects of social life, questioning simplistic views of cultural homogenization, probing into the identity and transformation, politics and democracy, and the nature of modern experience in translating, this book updates our comprehension of the global characteristics of contemporary society from the perspective of translation study.

With the acceleration of globalization and strengthening of cultural integration and multilingual communication, the translational sociology has opened a new pathway to address the increasing global challenges in and outside the societies of all countries. Of which, it is necessary to challenge the universal concepts that take language homogenization for granted, and move toward a multilingual vision. By turning our attention to the ever-expanding differences and transformation through inter-language communication in translation study, this book presents us with more extensive social context, away from the silence of polyglots to the pleasures of bilingual games, which in many ways provides an unexplored field for sociological translation. In this book, Bielsa (2023) puts emphasis on the call for reflexive translation as a form of translation, argues to extend this practice to all translation users, so that we can become the reflexive authors of the translation, some practical approaches of this interdisciplinary efforts are presented. The research reveals distinctive traits like (1) An association of translation with movement and transformation; (2) An attention to the key intervention of local actors and to spaces of contestation and resistance to the global diffusion of practices and norms; (3) A broad view of translation as relating not just to texts but to emerge social relations between previously unconnected people, materials and things; and (4) A critical call to rethinking their disciplines through translation (Bielsa, 2023, p. ii).

This book contains three parts divided into nine chapters, with notes and references for each chapter, and a bibliography, an index in the end of the book. Part 1 (Chapters 1–3) demonstrates arguments on the relationship between translation and identity (Adorno, 2004), transformation for Translational Sociology in detail. It helps readers obtain more systematic, comprehensive and dialectical understanding of the basic concepts and development process of Translational Sociology, get access to how the translation activity interacts with political and cultural life in practice. Chapter 1 introduces the core theories, main issues, and applications of translation and identity in modern society, including the discursive explosion of identity, critical issues, and debates on strangeness of translating. Chapter 2 works on translation and transformation by the discussion on the similarity between sociology and translation studies, tending to highlight continuity rather than change. Additionally, a specific analysis of two sociological approaches to metamorphosis, i.e., metamorphosis and/of knowledge beyond disciplinary boundaries, translation and metamorphosis, to elaborate how the concept of metamorphosis is related to the current major challenges, and how the view of translation as metamorphosis can help clarify and search for answers to these challenges (p.43). Chapter 3 explains four aspects of translation (p.56) to reinforce the framework of translational sociology. The author not only challenges the belief of automatic translation, argues why sociology needs translation, but also expounds the central significance of translation to society, and identifies four crucial ways in which translation shapes social reality and the essence of sociological efforts (p.69).

Part 2 (Chapters 4–6) introduces new terms such as politics, democracy in translating by exploring the interaction between translation and politics in three aspects: politics of translation, translating democracy, and the translator as producer. Chapter 4 presents six points of view in a speech of translation and revolution, i.e., the reasons for the urgent need for translation politics in today’s society; questioning the current definition of translation; an overview of how ethnocentrism has become the central tendency or resistance in any translation act; the explanation of a politics of translation of openness to the other on the basis of idea of hospitality (Derrida and Dufourmantelle, 2000, p.81); the relationship between translation politics and identity; the political and social relevance of translation in the contemporary context. Chapter 5 discusses how the debate on language and democracy is conducted within the framework of multiculturalism and the world, raising questions on some basic assumptions, proving the basic continuity with the vision of a single language, which consequently proposes an alternative concept of democratic language based on multilingualism, language hospitality, and translation. Chapter 6 analyzes and displays the methods that translators can better perform translation as work as producers (Venuti, 2008) from four fields: moving authors and readers home and abroad; interdisciplinary approaches to translation as transformation, politicizing translation; assimilatory and reflexive translation as an outline, which attempts to promote the task of reexamining the current discipline direction by rethinking the politics of translation from an essentially interdisciplinary perspective.

Part 3 (Chapters 7–9) highlights translation and experience. Chapter 7 concentrates on Walter Benjamin’s approach to the experience of modernity through his prolonged participation in poetry of Baudelaire (1988), and the description of three aspects of translating Baudelaire: (1) translating Baudelaire: articulating the task of translation; (2) interpreting Baudelaire: the experience of modernity; and (3) recovering a philological attitude (pp.116–129). By the analysis of the above, Chapter 7 reveals the relationship between Baudelaire’s translation and interpretation in work of Benjamin (1996), trying to restore the systematic nature of his thoughts, and break away from the traditional disciplinary boundaries and the traditional differences between translation, writing, and criticism. Chapter 8 clarifies a view of the cosmopolitan stranger centered on the stranger’s subjective experience (Millan, 2006) of the foreign (p.146), in which the first two sections reconstruct the sociological theory of strangers and conceptualize the cosmopolitan strangers; the last two sections make two case studies. Chapter 9 reviews the author’s experience of returning home in the past 10 years from different angles and times by three articles, the first (2012) tells the experience 2 years after the author returned to Spain, under the collective mobilization of researcher Ramon y Cajal (RyC); the second (2019) was to celebrate 50 years of Sociology, Anthropology and Criminology at Glasgow University, where the author did part of her undergraduate degree and her PhD; the last reflects on the author’s experience of securing tenure through the Spanish system of competitive examinations.

One of the main values of this book is, it has effectively demonstrated why translation is so important for the command of the mate modern reflexive imperative by Margaret Archer, which makes us become more reflexive in our daily conversations with ourselves and others (Archer, 2012). However, this is not the only crucial element because of the fast-paced structural variations and the growing unreliability of social actors to tacit knowledge and habits. In the context of cultural integration and multilingual communication, it is necessary to challenge those common concepts that take language homogenization for granted, and move toward a multilingual vision to meet the increasing global challenges within and outside societies. Moreover, this book calls for a corm of translation that is becoming increasingly relevant in our society—reflexive translation. It is necessary to promote the practice of reflexive translation to all translation users to make it more radical, so as to provide us with an approach to rethink the language heterogeneity we usually involve, and let us become the reflexive author of the translation we employ to understand the world. This is bound to be an interdisciplinary effort, involving the humanities and social sciences, moving toward an unexpected change, in believing sociology is in a favorable position to formulate and lead in this transformation, which not only affects sociologists as users of translation, but also the producers of translation.

One of the most constructive idea in understanding translation and identity by the author is that identity helps to actively re-recognize the social particularity of individuals and groups in a complex and heterogeneous society, whereas the most obvious defects of identity are the assumption of group boundedness and the ready-made matching between individuals and groups as its basis. In the context of globalization of strangeness nowadays, the problem is more prominent. This means the realization of globalization has not only led to the enhancement of people’s awareness of a smaller and highly interconnected world, but also led to the increase of people’s sense of strangeness. That could be why Rumford (2008) studied the proliferation of alien space in a world increasingly regarded as uncertain and threatening, as well as the blurring and reconstruction of borders at the national and global levels. He described strangeness as a more general experience of globalization, which in many ways makes the social world unrecognizable, and defined it as a social disorientation. Based on this, the author proposes that the major shortcomings of identity can be overcome by emphasizing non-identity and paying attention to what is missing from identity rather than affirming it.

In addition, the author argues that translation plays a vital role to conceptualize the above view based on contradiction and transformation rather than unity and the same cross-cultural relationship, also promotes to clarify an alternative view that does not exclude the particularity of the universal view. Furthermore, “cultivating vigilance and sensitivity to the complex translation process will help deepen sociological understanding of the meaningful nature of social life and self-reflexive engagement with sociological traditions” (p.20). This new concept endows translation and sociology with new mission and significance.

With the growing awareness of the importance of multilingualism and translation in crucial aspects of world projects, such as global democracy (Archibugi, 2008), human rights (Santos, 2010), transnational or cosmopolitan citizenship (Balibar, 2006), social movements (Santos, 2005), and borders (Balibar, 2010), how to grasp the potential epistemological issue of diversity within the world has become one of the vital ones in translation research. Inspired by Santos (2010), the author takes a whole chapter to introduce the necessity and specific content of formulating translation politics in detail, providing clear and detailed theoretical support for the practical application of translation. In discussing the relationship between ethnocentrism and translation, Beck (2006, p.89) and Delanty (2009, p.13) agree with the simple assumption of the possibility of transcending ethnocentrism through translation. The author (p.69) questioned their hypothesis and called on people to pay more attention to the definition of translation as a kind of ethnocentric violence, for emphasizing that what is interesting about translation is the struggle against the establishment of cultural ethnocentrism in any translation act, which has pushed forward obviously the notion proposed by Mona Baker in 2006.

In the understanding of the democracy of translation, unlike Will Kymlicka’s view that democratic politics is dangerous and even reactionary in vernacular (Kymlicka, 2001), the author believes that “multilingualism and translation do not harm democracy, nor are they the reserves of privileged intellectual elites, but are the sources of much-needed incentives”(p.89), which allows us to keep a distance from uncensored beliefs according to the differences of others, participate in the democratic decision-making between strangers, and stress the importance of replacing monolingual vision with multilingual vision. It argues that “translation enables strangers to face and effectively solve the tension and contradictions that inevitably arise when people of different races need to find a way to live together and collectively solve common problems” (p.89). Moreover, with the increasing status of translation in the humanities and social sciences, the essential role of the discipline of translation studies has become dramatically distinct. Based on this, the new concepts proposed by the author—assimilatory translation and reflexive translation—have also been adopted to directly tackle the major disputes in the social sciences about the contemporary social reality. In addition, the author points out that a key contribution of translation studies is to reveal the central role of language conversion process in any translation concept. As far as the translation turn is concerned, it also includes the criticism and rethinking of the basic discipline directions and concepts that have been systematically ignored or taken for granted. “Reflexive translation can also become the key interdisciplinary practice in this direction” (p.108).

In order to shed light on the relationship between translation and modernity, the author tries to experience modernity by introducing Walter Benjamin’s participation in Charles Baudelaire’s poetry. The author (p.115) believes that the translation scholars’ study of The Task of the Translator has not linked it with Benjamin’s interpretation of Baudelaire, or even thought about how Benjamin’s thought has undergone great changes in the later years, these changes have had an impact on the interpretation of this early work. Sociologists focused on Benjamin’s interpretation of Baudelaire in order to express modern experience and modernity theory, but did not stop to reflect on how Baudelaire’s experience in translation had affected this interpretation. At the same time, the author believes that *The Age of Translation* of Berman (2018, p. 32) is a long review of Benjamin’s key articles, not considering how Benjamin’s interpretation of Baudelaire is related to the potential systematic nature of Benjamin’s fractured writing. But the author expounds the basically consistent view of the social significance of language and translation in Benjamin’s entire intellectual trajectory, and draws the reader’s attention to the generally underestimated aspects of Benjamin’s modern experience theory, because the existing academic emphasis on the graphic and visual elements is more related to dialectical images, rather than Benjamin’s language method. This is a key contribution to the sociology of translation, because it supports a non-reductive approach to the linguistic diversity in social life, reveals the relationship between Baudelaire’s translation and interpretation in Benjamin’s works, on the purpose of restoring the systematic nature of his thoughts to break away from the traditional disciplinary boundaries and the traditional differences between translation, writing and criticism.

An innovative discussion in the book about translating strangers is, the author summarizes the characteristics of the cosmopolitan stranger as follows: first, cosmopolitan strangers must be theorized, between cosmopolitans and contemporary strangers. The former is home in all parts of the world, and the latter is eternal vagrant, “homeless always and everywhere, without hope of ever arriving” (Bauman, 1991, p.79). Second, the cosmopolitan strangers are famous for their excellent translation techniques. Third, the cosmopolitan stranger is a mediated stranger, which is becoming increasingly important not only in face-to-face interaction with a close group, but also in the broad and heterogeneous public. Fourth, the cosmopolitan strangers are not the only, or even the most common strangers nowadays. The author also proposes a concentration on the worldwide social ability of immigrants, emphasizing the simultaneous rooted and open, as well as cultural differences, as a source of personal creativity to adapt and build new homes in different environments (*cf.* Beck, 2006, p. 104; Sennett, 2009; Glick Schiller et al., 2011; Agier, 2016).

In summary, this book has systematically and comprehensively argued the identity, democracy, and modernity from interdisciplinary perspective of translation study, cultural study and sociology, making efforts to construct a stronger bridge to go through translation and sociology. The new concepts of assimilatory translation and reflexive translation put forward by this book would bring some further innovation and inspiration to the traditional idea of functional equivalence and discussion of translation technique, method, and strategy. However, the research scope can be enlarged to a kind of wider synchronic survey, such as more examples from Asian societies, more data from the oriental literary works, political discourses to be cited to make comparative study, and giving more comprehensive insights to translation sociology to avoid Eurocentrism when the discussion of ethnocentrism was made. In spite of this, the book has pushed forward further both study and practice of translational sociology and settled down new model of the future translation study.

## Author contributions

JX and CL proposed the idea. JX and YB made the first draft of the manuscript. JX and CL revised and finalized the manuscript. All authors contributed to the article and approved the submitted version.
